# Optimization and Molecular Simulation of Gelatin‐Free Marshmallow Formulation Using Gellan Gum and Aquafaba

**DOI:** 10.1002/fsn3.71898

**Published:** 2026-05-24

**Authors:** Elif Ersahin Telli, Serpil Pekdogan Goztok, İbrahim Palabiyik, Deniz D. A. Kamer, Nevzat Konar

**Affiliations:** ^1^ Food Engineering Department, Agricultural Faculty Namik Kemal University Tekirdağ Turkiye; ^2^ Department of Food Processing Programme, Vocational School of Technical Sciences Siirt University Siirt Turkiye; ^3^ Dairy Technology Departmant, Agriculture Faculty Ankara University Ankara Turkiye; ^4^ Institute of Food Safety Ankara University Ankara Turkiye

**Keywords:** aquafaba, gelatin‐free, gellan gum, molecular dock

## Abstract

This study aimed to optimize the formulation of marshmallow‐type products by replacing animal‐derived gelatin with plant‐based aquafaba and gellan gum. The formulation optimization was conducted using response surface methodology, with the optimal formulation determined based on the density and overall preference. In the optimized formulation, the inclusion rates of aquafaba and gellan gum as gelatin substitutes were determined to be 0.71% and 0.50%, respectively. The marshmallow samples were compared to control marshmallow samples containing gelatin (CM). The interaction between aquafaba and gellan gum was further investigated using molecular docking. The most stable predicted molecular configuration yielded a binding energy score of −9.8 kcal/mol, suggesting a favorable interaction. The water activity, density, and *T*_end_ values of the optimized formulation (OFM) samples were 0.56, 0.84 g/cm^3^, and 81.65°C, respectively, all of which were higher than those of the CM samples. A significant difference was observed in the color values between the OFM and CM samples, despite no colorants being used in either formulation (*p* < 0.05). The PLM images revealed that the granule dimensions in the CM samples were larger and more prominent compared to the OFM samples. The OFM samples also exhibited higher viscosity and superior viscous rheological properties compared to the CM samples.

## Graphical abstract

This study optimized a novel, gelatin‐free marshmallow formulation using plant‐based aquafaba and gellan gum, demonstrating thermodynamically favorable protein interactions and viable physical properties. These findings highlight the potential of this hydrocolloid complex as a sustainable alternative to animal‐derived gelatin in aerated confectionery products.

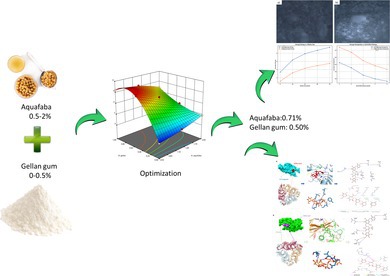

## 1 Introduction

Confectionery products are among the most popular foods worldwide, appealing to a broad consumer base (Pekdogan Goztok et al. 2022; Gunes et al. 2022). These products encompass a wide variety, offering almost limitless variations. Marshmallows, a type of soft confectionery, stand out as a unique product group renowned for their foamy texture, which garners significant interest globally. With a distinctive viscous consistency and remarkable sensory properties influenced by aroma and coloring agents, marshmallows also possess a chewy, finely porous foamy structure (Ghendov‐Moșanu 2018; Piliugina et al. 2019; Covaliov et al. 2021). The characteristic foamy structure of marshmallows results from the incorporation of air into the product through the rapid mixing of a protein and sugar mixture. This aeration process not only increases the mixture's volume but also enhances its softness, creating a lighter texture. It alters the product's rheological and visual properties, intensifies flavor perception, and significantly improves digestibility due to increased porosity (Periche et al. 2016). Additionally, while a prolonged shelf life is often associated with low water content, the volume increase from aeration also contributes substantially to preserving shelf life (Eddy and Editya 2020).

Gelatin, the primary protein used in marshmallow production, performs two essential functions: it serves as an excellent foaming agent and an effective stabilizer. During aeration, gelatin enables the sugar syrup to develop specific elastic characteristics, resulting in the formation of a white, foam‐like air mass. However, despite its functional benefits, gelatin use is undesirable for various consumer groups due to dietary restrictions or ethical considerations (Samira 2018). Gelatin is predominantly produced from animal sources—80% from pig skin, 15% from cow skin, and 5% from cattle bones. Its production is steadily increasing to meet consumer demand. Nevertheless, gelatin consumption raises ethical and dietary concerns for specific consumer groups such as vegetarians, Jews, and Muslims (Sani et al. 2021). Additionally, the use of gelatin poses potential health risks due to the possibility of contamination with pathogenic microorganisms. These issues have prompted research into alternative gelling agents including patents for fish‐based gelatin production and substitutes derived from hydrocolloids such as starch/modified starch, pectin, carrageenan, and agar (Karim and Bhat 2008). In the confectionery industry, there is growing interest in developing gelling agents of plant, microbial, or polysaccharide origin. These alternatives, whether used alone or in combination with other polysaccharides, require scientific validation to assess their suitability as gelatin substitutes.

Studies on hydrocolloids or similar materials as alternatives to gelatin, particularly for marshmallow production, have shown promising results. For instance, Mardani et al. (2019) used xanthan gum and guar gum in combination. Their findings indicated that substituting gelatin with xanthan and guar gum increased the moisture content and water activity of the samples. They concluded that these hydrocolloid mixtures could effectively replace gelatin in marshmallows, provided the appropriate mixture and ratio are used to achieve acceptable aeration and foaming properties. Similarly, Sheng et al. (2016) investigated foam formulations containing xanthan gum and found that while foam stability improved with the addition of xanthan gum, its foaming ability remained largely unaffected. These findings underscore the potential of hydrocolloids as viable gelatin substitutes in confectionery products.

Gellan gum is widely utilized in the food industry as a gelling, thickening, and binding agent in products such as confectionery, creams, jams, and jellies. It is also used as a gelatin substitute in dairy‐free products like vegan yogurt and cream (Jindal and Khattar 2018). Its liquid gel‐forming capability makes it suitable for applications in puddings and broths. Additionally, combining fish gelatin with gellan gum has increased its potential use in the gel industry (Yilmaz et al. 2023). Gellan gum is highly functional at low concentrations compared to other gelling agents and can form gels with various ions (Sworn et al. 1995). It serves as an alternative to pectin in jellies, which typically require high sugar levels for gel formation (Dev et al. 2022). Furthermore, gellan gum has been combined with carrageenan and starch in solid confectionery products to enhance elasticity and translucency (Karim and Bhat 2008).

Increasing consumer awareness of environmental issues has led to a shift toward plant‐based, environmentally friendly foods to reduce the carbon footprint and address climate change concerns (He et al. 2021). Aquafaba, a viscous liquid typically regarded as a byproduct or waste, is obtained by cooking legumes such as chickpeas and beans, which form a significant component of traditional diets (Tufaro and Cappa 2023). During the cooking process, water‐soluble polysaccharides and proteins leach into the water. Aquafaba is composed of approximately 95% moisture, 1% protein, and 1.3% soluble carbohydrates including sugars. It is also rich in saponins and phenolic compounds (Raikos et al. 2020).

He et al. (2021) identified aquafaba as a novel plant‐based rheological additive, highlighting its foaming, emulsification, and gelling properties. The literature documents various uses of aquafaba as an egg substitute including in cakes (Nguyen et al. 2020; Aslan and Ertaş 2020), sponge cakes (Mustafa et al. 2018), and vegan mayonnaise (Raikos et al. 2020). Studies have shown that the foaming stability of cakes containing aquafaba is comparable to that of cakes made with eggs, supporting its viability as an egg replacement. Given its physical and chemical composition, quality, and functional properties, aquafaba has significant commercialization potential. Its use as an emulsifier, foaming agent, or binding agent is suitable for a wide range of products including mayonnaise, cakes, salad dressings, bread, meringues, cheese, ice cream, and confectionery.

Molecular docking is a computational technique extensively used in structural biology and drug discovery to predict the preferred orientation of a ligand as it binds to a protein's active site. This method evaluates the interactions between the ligand and the protein, providing insights into binding affinities and the most energetically favorable conformations (Morris et al. 2009). In this study, protein‐ligand complex interactions between major proteins in aquafaba and gellan gum were analyzed using molecular simulation.

This research utilized aquafaba, known for its foaming properties, and gellan gum, valued for its gelling capabilities, as substitutes for gelatin. The study was conducted in two stages. The first stage involved determining the optimum composition of gelatin‐free marshmallow samples based on density and sensory analysis results from the experimental design. The second stage focused on comparing the quality characteristics of the optimized marshmallow sample with those of a gelatin‐containing marshmallow.

## 2 Materials and Methods

### 2.1 Materials

In the preparation of marshmallow‐type soft confectionery samples, the following ingredients were used: water, powdered sugar (TSM Food Industry Trade Corporation), 250‐bloom bovine gelatin (Sel‐Gel Gelatin, Istanbul), gellan gum (CP Kelco, Kelcogel Gellan Gum), and aquafaba powder (Döhler, Istanbul).

### 2.2 Study Model

A Response Surface‐Central Composite Design (CCD) was employed using the statistical software package Design‐Expert (Stat‐Ease Inc., version 7.0, Minneapolis) to determine the optimal proportions of two independent variables in the model system. The design consisted of 10 experimental points. The effects of aquafaba (*X*_1_) and gellan gum (*X*_2_) concentrations on the physicochemical, color, texture, differential scanning calorimetry (DSC), polarized light microscopy (PLM), and sensory properties were evaluated, leading to the determination of the optimal combination.

The study design and ingredient proportions are presented in Table 1. Based on preliminary tests, the minimum and maximum concentration levels for aquafaba and gellan gum in the marshmallow samples were set at 0.5%–2% and 0%–1%, respectively (Table 1). For optimization, the combination of factors that provided the most favorable responses was determined, considering the influence of each variable.

**TABLE 1:** Central composite mixture design study model and sample preparation.

Run	Coded values		Real values		Other values		Total
	*X*_1_	*X*_2_	Aquafaba (%)	Gellan gum (%)	Sucrose (%)	Water (%)	
1	1.25	1.00	1.25	1.00	68.00	29.75	100
2	1.25	0.50	1.25	0.50	68.00	30.25	100
3	2.00	0.50	2.00	0.50	68.00	29.50	100
4	2.00	1.00	2.00	1.00	68.00	29.00	100
5	2.00	0.00	2.00	0.00	68.00	30.00	100
6	0.50	0.00	0.50	0.00	68.00	31.50	100
7	1.25	0.00	1.25	0.00	68.00	30.75	100
8	0.50	1.00	0.50	1.00	68.00	30.50	100
9	1.25	0.50	1.25	0.50	68.00	30.25	100
10	0.50	0.50	0.50	0.50	68.00	31.00	100

*Note:* *X*_1_, aquafaba; *X*_2_, gellan gum.

### 2.3 Sample Preparation

The marshmallow sample containing gelatin was prepared following the method described by Pekdogan Goztok et al. (2022). Initially, 27.1 g of water per 100 and 66 g of powdered sugar per 100 g were mixed in a thermal mixer (Thermomix TM5; Vorwerk, Wuppertal, Germany). The mixture was heated to 107.0°C ± 3.00°C at 200–300 rpm until reaching a concentration of 88 °Brix. The mixture was then rapidly cooled to 70°C, after which a 250‐bloom gelatin: water solution (1:2) was added and mixed for 10 min at 200–300 rpm, maintaining a temperature of 70°C. Whipping was performed at 45°C–55°C and 200 rpm for 10 min. For the preparation of gelatin‐free sample groups, aquafaba powder was first dissolved in water at a ratio of 1:10 (aquafaba/water) and whisked at 700 rpm for 40 min. In parallel, powdered sugar and water were mixed and heated at 107.0°C ± 3.00°C at 300 rpm for 10 min. Gellan gum was then added to the sugar syrup and mixed for an additional 5 min. The prepared aquafaba foam was gradually added to the sugar syrup containing gellan gum, and aeration was performed at 200 rpm for 10 min while cooling the mixture to 40°C. The final mixture was poured into silicone molds and allowed to set at 15.0°C–18.0°C for 24 h. The optimum composition for the gelatin‐free samples was determined using the Design‐Expert program, considering the density analysis results and sensory evaluation for general appreciation of the marshmallow samples. Marshmallow production was carried out using the optimized formulation, and the results of various quality analyses were compared with those of the control sample.

### 2.4 Water Activity Analysis

The water activity (*a*_w_) of the marshmallow samples (2 g) was determined using a Lab‐Master *a*_w_ (Decagon AquaLab, 4 TE) at 20°C–25°C according to the method used by Gok et al. (2020). The *a*_w_ value of each sample was determined in triplicate.

### 2.5 Density Analysis

The density of the samples was determined by the method used by Mardani et al. (2019). Marshmallow samples were transferred to a container of known volume (100 mL) and then the foam mass weight was determined. The density was calculated using the following equation (Equation 1):

(1) $$\text{Density}=\text{Foam mass}\;\left(g\right)/\text{volume of container}\;\left(\mathrm{mL}\right)$$

### 2.6 Color Analysis

Color parameters of marshmallow samples, L*: brightness, a*: ±red–green, and b*: ±yellow–blue were determined using a colorimeter (Chroma Meter CR‐400; Konica Minolta, Japan) (Periche et al. 2015). The analyses were performed in five replicates. Chroma (C*), and hue (h*) values were calculated using Equations (2) and (3):

(2) $$C*=\sqrt{a{*}^{2}+b{*}^{2}}$$

(3) $$h*=\arctan\left(b*/a*\right)$$

### 2.7 Differential Scanning Calorimetry (DSC)

The glass transition temperatures of marshmallow samples were determined by using differential scanning calorimetry (TA Q20; TA Instruments, New Castle, USA), and an empty pan was used as a reference. Samples (~5 mg) were weighed in aluminum pans and hermetically sealed with lids by using a sample press. Pans were heated at 5°C/min from 5°C to 90°C in a nitrogen atmosphere (Atik 2021). Each sample was analyzed in duplicate.

### 2.8 Polarized Light Microscopy (PLM) and Microstructural Analysis

A polarized light microscope (Zeiss Carl, NY, USA) which is equipped with a heating–cooling system was used to observe the melting properties of the marshmallow samples. Photographs were taken using ZEN core v3.1 (Carl Zeiss Microscopy LLC, NY, USA) software (Atik 2021). The images were selected from random areas of the samples and at least 3 images were taken from each sample. A Gray‐Level Co‐occurrence Matrix (GLCM)‐based analysis method was used to quantitatively determine the microstructural texture properties of the materials and to compare the differences between the samples. These analyses were conducted in the Google Colab cloud computing environment using the Python programming language and the scikit‐image library that were utilized for this analysis. The change in entropy and homogeneity values depending on distance has been presented graphically to show the spatial variation in the structural complexity of the samples.

### 2.9 Molecular Docking Analysis

The protein‐ligand complex interactions between major proteins in the aquafaba structure and gellan gum were investigated using molecular simulation. The docking simulation incorporated AutoDock Vina (Pyrex; https://pyrx.sourceforge.io), Discovery Studio (https://www.3ds.com/products/biovia/discovery‐studio), and the UCSF Chimera program (University of California, San Francisco, CA, USA) as virtual screening software. Aquafaba's protein content is primarily composed of 11S legumin and 7S vicilin. For the simulation, the crystal structures of 11S legumin (PDB ID: 3KSC, Resolution: 2.86 Å) and 7S vicilin (PDB ID: 7U1I, Resolution: 3.00 Å) were selected as they are well‐established, representative experimental structures for these chickpea proteins (Meng et al. 2023). Therefore, the interaction between these two representative proteins and gellan gum was simulated. Protein data were obtained from the Protein Data Bank of the Research Collaboratory for Structural Bioinformatics, accessible at https://www.rcsb.org. Prior to docking, the protein structures were prepared using the UCSF Chimera program. This preparation involved several steps: all water molecules and co‐crystallized ligands were removed, polar hydrogen atoms were added, and Kollman charges were assigned. Any missing residues in the original PDB files were repaired using the program's built‐in tools. A description of the ligand structure (gellan gum) was performed using ChemDraw. The docking simulation incorporated AutoDock Vina within the PyRx virtual screening tool. For the simulation, a grid box with dimensions of 70 × 70 × 70 Å was defined to encompass the active binding site of each protein. The center coordinates for the grid box were set to *x* = 41.454, *y* = 245.047, *z* = 58.119 for 11S legumin (3KSC) and *x* = 22.233, *y* = −23.352, *z* = −12.484 for 7S vicilin (7U1I). The exhaustiveness, which controls the thoroughness of the search, was set to 8. A description of the ligand structure (gellan gum) was performed using ChemDraw. PyMOL software was utilized for the binding site visualization. Proteins Plus (DoG Site Scorer) (https://proteins.plus/) and the PLIP tool (https://plip‐tool.biotec.tu‐dresden.de/plip‐web/plip/index) were used to create the 2D and 3D diagrams, respectively.

### 2.10 Rheological Properties

The viscoelastic properties of marshmallow samples were determined by Discovery HR‐2 Rheometer (TA Instruments, New Castle, USA). Frequency scans (mechanical spectra) were performed to explain the time‐dependent behavior of the samples at a gap of 1200 μm in a 40 mm diameter parallel plate and a Peltier plate. The angular frequency range was chosen between 0.1, 100, and 1 rad/s, and the strain value was applied as 0.01%. The temperature was controlled at 25°C ± 1°C using a Peltier system. The Herschel‐Bulkley model was used to measure the flow ramp values of the samples.

Herschel Bulkley model (Bourne 2002)

$$\sigma =\sigma_{0}+{\left(\dot{\gamma }\right)}^{n}$$

where *σ* denotes the shear stress (Pa), *σ*_0_ denotes the yield stress, *γ* is the shear rate (s^−1^), *K* is the consistency index (Pa.s^n^), and *n* denotes the flow behavior index (dimensionless).

### 2.11 Texture Analysis

Texture profile analysis (TPA) of the marshmallow samples (28Ø × 20 mm) was performed using a TA. XT Plus Texture Analyzer (TA‐XT; Stable Microsystems, New Castle, USA) equipped with a load cell of 5 kg and a P/36 cylindrical probe. Test parameters was pre‐test speed 2.00 mm/s, test speed 5.00 mm/s, post‐test speed 5.00 mm/s. From the resulting forcetime curve, the following parameters were quantified; hardness (N), gumminess (g), springiness, cohesiveness and resilience (Altınok et al. 2020). Each measurement was carried out with five replications.

### 2.12 Sensory Analysis

Sensory analysis was conducted with 20 untrained panelists (12 female, 8 male, ages 20–45), who were regular consumers of confectionery products and familiar with marshmallows. Marshmallow samples, cut into uniform sizes (30 × 20 mm), were presented on white plates. Each sample was coded with a random three‐digit number, and the order of presentation was randomized for each panelist to avoid order bias. The sensory characteristics of marshmallow samples (appearance, flavor, texture, mouth melting rate, adhesiveness, and overall liking) were determined using a 9‐point hedonic scale (1 = not at all like, 9 = very much like). The evaluation was conducted under a controlled room temperature of 25°C ± 1°C.

### 2.13 Statistical Analysis

Evaluation of the analysis results was carried out using ANOVA variance analysis. Pearson correlation coefficient (*R*), which gives the possible correlation between the quality parameters of marshmallow samples, was performed using Design Expert (Stat‐Ease Inc. version 7.0, Minneapolis). The variances were considered statistically significant at *p* < 0.05.

## 3 Results and Discussion

### 3.1 Density and Sensory

**FIGURE 1:**
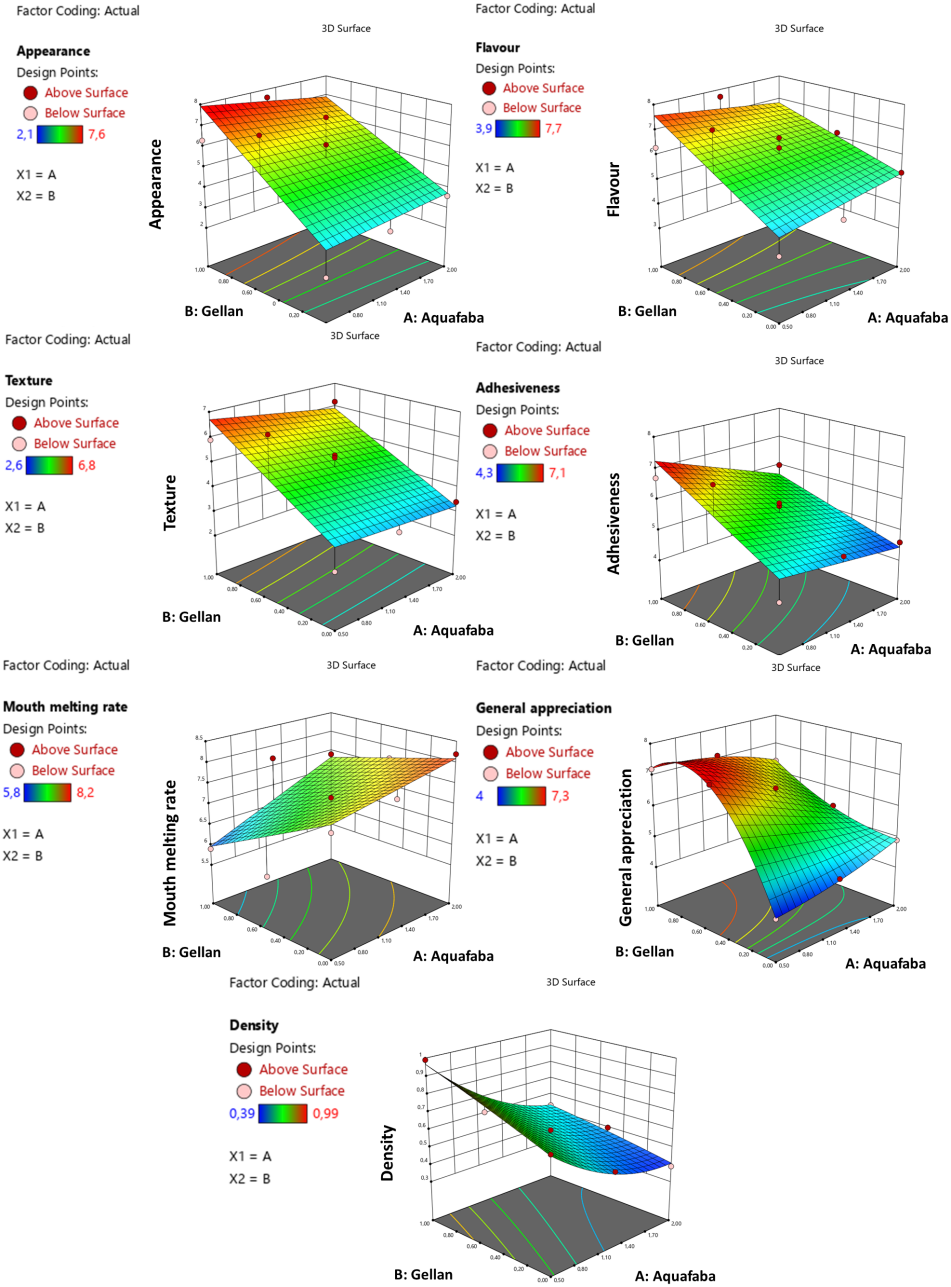
The result of sensory analyses (appearance, flavor, texture, adhesiveness, general appreciation) and density of gelatin‐free marshmallow samples.

The sensory properties affect individuals' desire to purchase as well as confectionery products. It is one of the critical factors that affects the quality (Şahin 2021). In order for sensory properties to be perceptible and evaluable, it is necessary to examine them by taking into account two or more stimuli (Erdem et al. 2023). For this reason, sensory evaluation of marshmallow samples was carried out with parameters that can be perceived by the eye and intra‐oral senses. The data related to sensory properties are given in Figure 1. Appearance, flavor, texture and adhesiveness values of marsmallow samples were determined in the ranges of 2.1–7.6, 3.9–7.9, 2.6–6.8, and 4.3–7.1, respectively (Figure 1). It was determined that the increase in the use of gellan gum instead of gelatin caused an increase in the parameters of sensory properties (appearance, flavor, texture, adhesiveness, general appreciation). When the melt‐in‐mouth rate values, which are considered important criterion in marshmallow type confectionery products with foamy structure, were examined. It has been determined that the mouth melting rate increased with the increase of aquafaba ratio, but the melting rate decreased with the increase of gellan gum ratio. The main reason for this is that aquafaba provides a foamy structure to marshmallow products, whereas gellan gum gives hardness to the structure of marshmallow products.

Foam structure and stability of marshmallow have a significant relationship with panelists' general appreciation (Table S1). General perception was improved by reducing the amount of Aquafaba used to make a gelatin‐free marshmallow product. This is due to the fact that aquafaba has a distinct odor from chickpeas and has a yellow color, which becomes more pronounced as its use increases in the samples. Therefore, there was no parallelism between the general appreciation parameter and density values (Figure 1). In the optimisation process, the balance between density and general appreciation was achieved as follows: the sample with the lowest density value and the highest general appreciation value was selected as optimum.

The protein ratio in the protein‐polysaccharide complex is an important criterion for foam formation and stability (Pekdogan Goztok et al. 2022). Density values of marshmallow samples varied between 0.39 and 0.99 g/cm^3^. The increase in the amount of aquafaba in the gelatin‐free marshmallow samples caused a decrease in the density value by increasing foaming. In a study conducted on the foaming capacity of aquafaba obtained from chickpea juice together with xanthan gum, it was reported that the addition of aquafaba positively affected the foam characterization and could be a plant‐based foaming agent that replaces eggs in cake products (Crawford et al. 2023). Similarly, Buhl et al. (2019) reported that aquafaba is a suitable candidate to replace protein content in foods such as marshmallows, where air binding capacity is important. When the effect of gellan gum increase on density was examined, an increase in the density values of marshmallow samples was observed as the gellan gum concentration increased (Figure 1).

The correlation and regression coefficients of the model determined in the response surface analysis of gelatin‐free marshmallow samples with experimental data were given Table 2.

**TABLE 2:** The correlation and regression coefficients of the model determined in the response surface analysis of gelatin‐free marshmallow samples with experimental data.

Variable	Coefficients							*R* ^2^
	*X*_1_	*X*_2_	*X*_12_	*X*_1_^2^	*X*_2_^2^	*X*_1_^2^_2_	*X*_12_^2^	
Appearance	0.266	5.033	−0.933					0.654
Flavor	0.444	3.433	−0.933					0.572
Texture	−0.188	3.350	−0.333					0.714
Adhesiveness	−0.377	2.566	−0.666					0.7096
General appreciation	3.947	12.244	−4.777	0.222	−9.100	−0.622	5.200	0.984
Density	−5.544	0.335	−0.093	0.139	−0.025			0.987

*Note:* *X*_1_, aquafaba; *X*_2_, gellan gum.

### 3.2 The Optimization of Formulation Determination of Gelatin‐Free Marshmallow Samples

The optimum content of gelatin‐free marshmallow containing aquafaba and gellan gum was determined after the density and sensory analysis results were entered into the Design Expert programme. The minimum density value and the maximum values of general appreciation were taken into account when optimizing. The importance value for both parameters was chosen as +++. The formulation of the gelatin‐free marshmallow containing aquafaba and gellan gum and the control group marshmallow samples containing gelatin are given in optimum 3.

In the next steps of the study, marshmallow samples produced with the ingredients of these formulations were used (Table 3). The final optimized formulation parameters for marshmallow‐type products are detailed in Table 7.

**TABLE 3:** The formulation of optimum formulation marshmallow (OFM) and control marshmallow (CM).

Ingredients	Optimum formulation marshmallow (OFM) (%)	Control marshmallow (CM) (%)
Powdered sugar	68.79	66.0
Water	30	27.1
Gelatin	—	6.90
Aquafaba	0.71	0.00
Gellan Gum	0.50	0.00

Abbreviations: CM, control marshmallow; OFM, optimum formulation marshmallow.

### 3.3 Water Activity

Water activity refers to the presence of water in the current system of foods, which is useful for assessing the shelf life, microbial, and storage stability of food products (Sablani et al. 2007; Tan and Lim 2008; Subbiah et al. 2020). The binding of water to the sugar and protein structures is a significant factor in determining the water activity (Rahman 2019). Water activity value in marshmallow products varies between 0.60 and 0.75 (Hartel et al. 2018).

Water activity (*a*_w_) values of OFM are given in Table 4. According to the results obtained, the water activity value of the OFM sample was determined 0.84 ± 0.01; the CM sample was determined 0.75 ± 0.01. The use of aquafaba and gellan gum instead of gelatin in marshmallow products causes an increase in water activity value (*p* < 0.05). The utilization of a greater quantity of water in the preparation of the aquafaba solution in comparison to the gelatine solution resulted in the CM sample containing gelatine exhibiting a lower water activity than the OFM samples. A similar result was found by Mardani et al. (2019) in which xanthan and guar gum were used as gelatin substitutes in marshmallow production. Additionally, they reported in their study that water activity (0.74–0.80) values increased with the addition of hydrocolloids to the formulation.

**TABLE 4:** Physico‐chemical (water activity and density) and color properties of OFM and CM samples.

Sample	Water activity (*a*_w_)	Density (g/cm^3^)	L*	a*	b*	C*	h*
OFM	0.84 ± 0.01^A^	0.56 ± 0.01^A^	91.49 ± 0.22^A^	0.43 ± 0.01^A^	1.22 ± 0.04^A^	1.30 ± 0.04^A^	2.95 ± 0.32^A^
CM	0.75 ± 0.01^B^	0.41 ± 0.01^B^	90.66 ± 0.08^B^	0.39 ± 0.01^B^	1.08 ± 0.01^B^	1.15 ± 0.01^B^	2.75 ± 0.02^A^

*Note:* Different capital superscript letters show the statistical difference between samples (*p* < 0.05).

Abbreviations: CM, control marshmallow; OFM, optimum formulation marshmallow.

### 3.4 Density

Density values of marshmallow products have been reported in the range of 0.2–0.7 g/cm^3^ (Hartel et al. 2018). Density values of marshmallow samples containing optimum aquafaba and gellan gum as gelatin substitutes are given in Table 4. The density values of the optimized gelatin‐free and gelatin‐containing marshmallow products were determined as 0.56 ± 0.01 and 0.41 ± 0.01, respectively. Moreover, the density value of the CM sample was found to be lower than the OFM samples (*p* < 0.05). Because as the air intake increases, the density value decreases (Campbell and Mougeot 1999). An increase in air intake results in a proportional increase in the number of air cells within the foam structure, which in turn leads to a reduction in the interfacial area. Consequently, both air and water activity a significant role in the formation of marshmallows. The addition of air to the product increases its volume and improves its texture. The presence of water in the product affects its viscosity, and therefore the ease with which foam can be formed during the aeration process is dependent on the water activity of the product (Klinjapo and Krasaekoopt 2018). The density values obtained were parallel to the water activity values of the samples. In addition, the density values of OFM samples were determined to be between the density values of standard marshmallow samples given in the literature. In this way, it has been determined that a plant‐based protein source has a foaming capacity close to that of gelatin, which is an animal protein. Kazantsev et al. (2022) reported in their study on foam stability that the addition of pectin led to an increase in foam mass and a parallel increase in density values.

### 3.5 Color

The external appearance (color and optical properties) of foods is one of the most important parameters affecting the sensory perception of consumers (Hutchings 2011). The color analysis values of marshmallow samples containing optimum aquafaba and gellan gum as gelatin substitutes are given in Table 4. Although no colorant was used in the marshmallow samples obtained with the control group and the optimum formulation, a difference in color values was observed (*p* < 0.05). In OFM samples compared to the CM sample, the increase in L* (brightness) value is caused by gellan gum, whereas the increase in b* (yellowness) value is caused by the unique yellowish quality of plant‐based aquafaba (*p* < 0.05). Furthermore, the gelatin‐free marshmallow sample exhibited a brighter hue, which is believed to be attributed to the enhanced water‐binding capacity of gelatin in comparison to the aquafaba‐gellan combination. A similar result in *L*, *a*, and *b* values was determined in the C* (saturation) value, and the C* values of the OFM samples were determined to be higher than the CM samples.

### 3.6 Thermal Behavior

Thermal properties of foods depend on their physical state (amorph and crystalline) (Terashima 2022). Differential scanning calorimetry is a universal method that measures the heat flux entering or leaving the structure through physical transitions in food samples (Manuel et al. 2022). The glass transition temperature plays an important role in determining the quality and shelf life of marshmallow‐type confectionery products containing a high concentration of sugar. The *T*_0_ (°C), *T*_dead_ (°C), and Δ*H* (J/g) values of gelatin‐free and control group marshmallow samples are given in Table 5. In addition, the *T*_0_ value, *T*_end_ value, and Δ*H* (J/g) values of gelatin‐free marshmallow samples were found to be 50.70°C, 81.65°C, and 205.12 J/g, respectively.

**TABLE 5:** The glass transition temperature analysis of OFM and CM samples.

Sample	*T*_0_ (°C)	*T*_end_ (°C)	Δ*H* (J/g)
OFM	50.7 ± 6.88^A^	81.7 ± 4.71^A^	205.1 ± 25.8^A^
CM	52.8 ± 3.47^A^	57.4 ± 3.90^B^	0.58 ± 0.26^B^

*Note:* Different capital superscript letters show the statistical difference between samples (*p* < 0.05).

Abbreviations: CM, control marshmallow; OFM, optimum formulation marshmallow.

Although the use of aquafaba and gellan gum did not make a statistically significant difference on the *T*_0_ value of marshmallow samples, it made a significant difference on the *T*_dead_ value and Δ*H* (J/g) values (*p* < 0.05).

### 3.7 Micro‐Structure

Polarized light microscopes (PLM) are used to magnify samples by interacting with light, enabling detailed imaging (Xiao et al. 2020). The microstructure of food provides valuable insight into its textural properties, offering important information about the volume and texture of the product. It is also used to examine phase transitions, such as gelation, by analyzing the crystal structure in confectionery products (Atik 2021).

**FIGURE 2:**
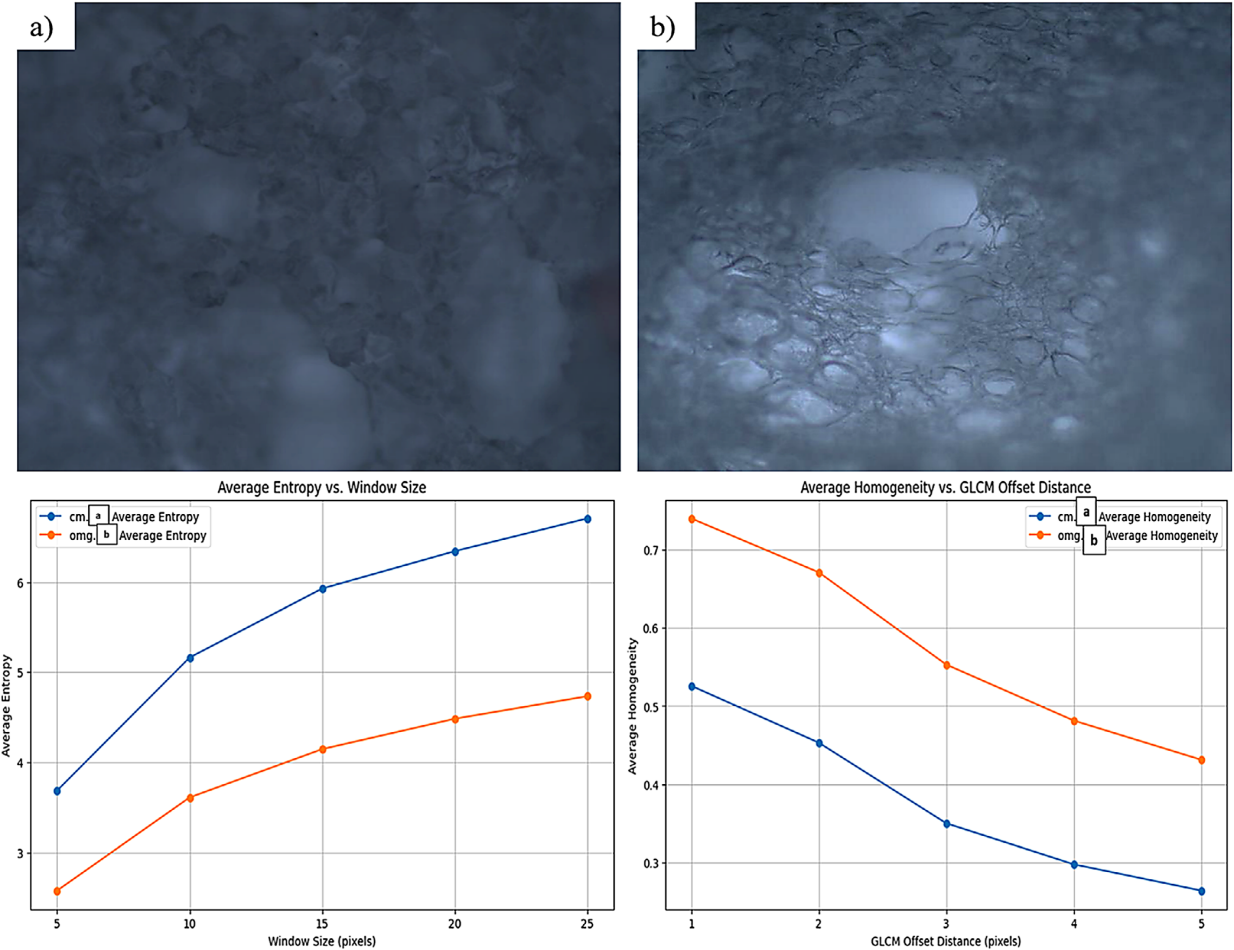
The polarized light microscopy images of OFM (optimum formulation marshmallow) and CM (control marshmallow) samples (×40): (a) OFM, (b) CM, and gray level co‐occurrence matrix (GLCM)‐based tissue analysis results.

In this study, the microstructure of both the gelatin‐containing marshmallow (CM) and the gelatin‐free marshmallow (OFM) samples was analyzed using polarized light microscopy. PLM images of randomly selected sections from the samples are presented in Figure 2. The results revealed that the granule diameters in the CM samples were larger and more pronounced compared to the OFM samples. Additionally, the boundary lines of the granules in the OFM samples were more distinct. This observed difference in granule structure may suggest a less stable foam in the OFM samples when compared to the CM samples. This visual evidence, combined with the higher density values of the OFM sample, could indicate a lower air retention capacity, potentially contributing to the observed differences in the foam structure. The PLM images suggest that the firm and stable foam structure characteristic of gelatin‐based marshmallows was not fully replicated with the aquafaba and gellan gum combination, which is consistent with the product's more spreadable consistency.

The Gray Level Co‐occurrence Matrix (GLCM)‐based tissue analysis performed demonstrates the microstructural differences between the CM and OFM samples. According to GLCM texture analysis results, the CM sample exhibited a more variable, rough, and heterogeneous microstructure with high Contrast (17.42) and high Difference (1.76) values. Low Homogeneity (0.53) and low Energy/ASM values indicate that pixel intensities frequently change in CM, suggesting an irregular pore distribution or small particles, thus supporting our claim that foam stability is reduced. In contrast, the OFM sample has a smoother, more uniform and regular texture with lower variability (0.78) and higher homogeneity (0.74) values (Figure 2).

### 3.8 Molecular Docking

**FIGURE 3:**
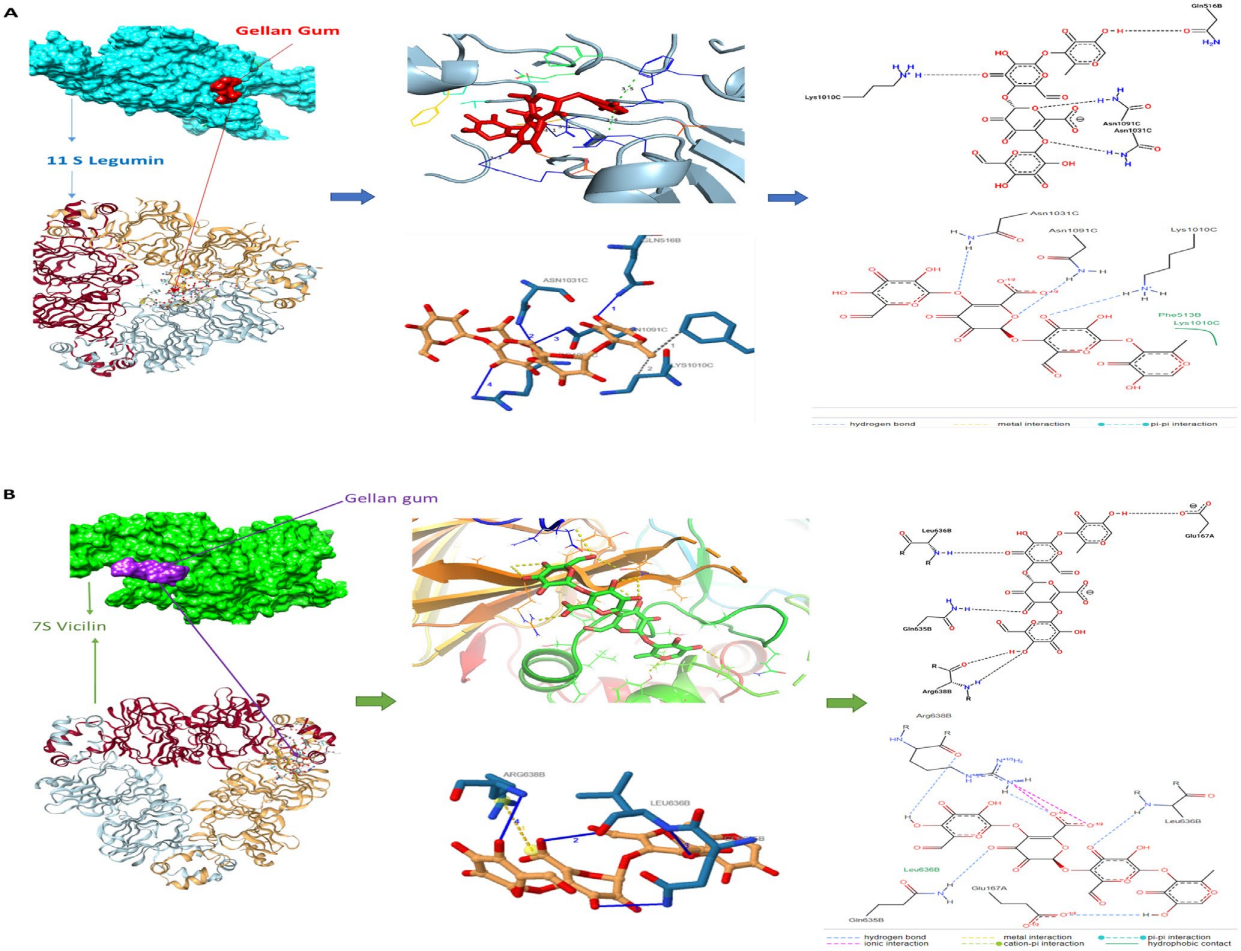
The molecular docking analyses of aquafaba and gellan gum.

**FIGURE 4:**
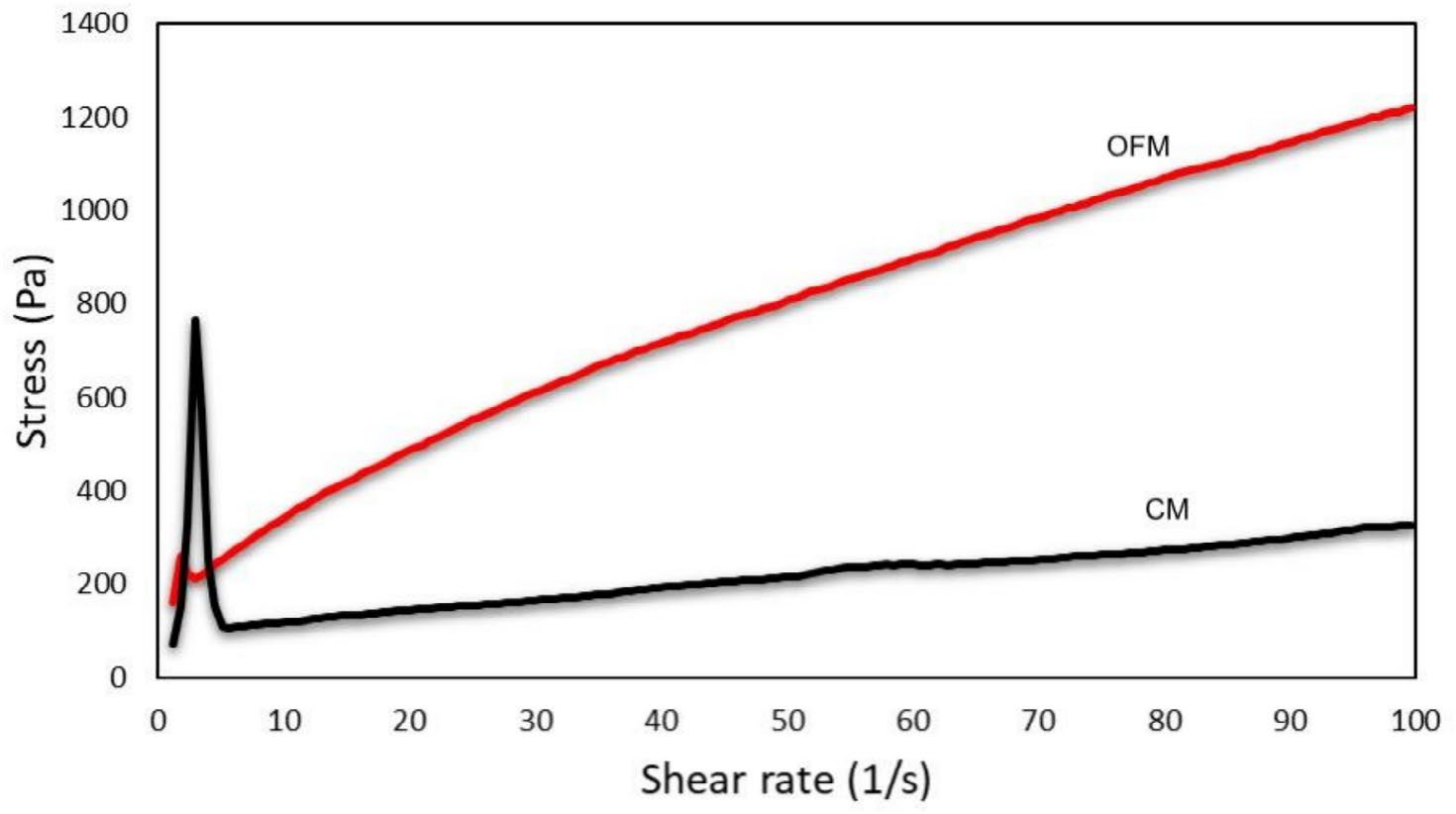
The rheological properties of OFM (optimum formulation marshmallow) and CM (control marshmallow) samples.

Molecular simulation has become a valuable tool for studying the interactions between macromolecule‐ligand complexes (Morris and Corte 2021). Molecular docking was used to investigate the interaction between aquafaba and gellan gum, and the resulting diagram of interactions is shown in Figure 3a,b. In docking algorithms, the most stable state is determined by the posture with the lowest binding energy or affinity because molecules have a thermodynamic tendency to exist in their lowest energy form, which has the largest entropy (Azam and Abbasi 2013). The docking simulation predicted a binding energy of −9.8 kcal/mol for the 11S legumin complex and −7.2 kcal/mol for the 7S vicilin complex. Although these AutoDock Vina scores are empirical estimates and not true thermodynamic free energies, their negative values suggest the interactions are thermodynamically favorable. The more negative score for the 11S legumin complex indicates a potentially more stable predicted conformation. The docking results revealed that the formation of all complexes occurred naturally. Figure 3a,b display the optimal conformation, the amino acid residues involved in the interaction, as well as the distances and forces involved in the binding of aquafaba 7S vicilin and 11S legumin. The 11S legumin interacted with gellan gum by hydrogen bonding (Gln516, Asn1031, Asn1091, Arg1093) and hydrophobic forces (Phe894, Lys1010). The amino acid residues Gln516, Asn1031, Asn1091, Arg1093 of 11S legumin interacted with GG at distances of 2.89, 4.07, 3.19, and 3.27 Å, respectively. The presence of hydrogen bonds greatly enhances the specificity and stability of the interaction by facilitating direct and robust intermolecular forces between the polar groups of the amino acids and GG (Pauling and Corey 1951). The hydrophobic interaction between 11S legumin and GG involved two amino acids Phe894 and Lys1010 at a distance of 3.55 and 3.57 Å, respectively. Although hydrophobic forces are typically less strong than hydrogen bonds, they are still important in stabilizing the protein‐polysaccharide complex by reducing the contact of non‐polar residues with water, therefore enhancing the overall thermodynamic stability (Peinado et al. 2010). The 7S vicilin interacted with GG by hydrogen bonding (Gln635, Leu636, Leu636, Arg638) with distances of 3.05, 3.76, 3.15, and 3.10 Å, respectively. Moreover, 3.87 Å salt bridge interactions between Arg 331 and carboxylate (Ligand) were identified. Salt bridges' electrostatic character allows them to withstand disruption by water; this provides a stronger interaction than the partial charges involved in hydrogen bonding. Studies have indicated that salt bridges can greatly help to maintain the stability of protein structures, particularly in hydrophilic surroundings where water molecules might disturb weaker connections (Han et al. 2022). Molecular docking analysis predicted favorable interactions between the aquafaba proteins and gellan gum. The negative binding energy scores suggest that the formation of these complexes is thermodynamically favorable, providing a molecular basis for the observed structuring effects of these ingredients when combined. The extensive hydrogen bonding and salt bridges between aquafaba proteins and gellan gum likely create a tightly bound, intricate polymer network. Although this network is strong on a molecular level, it may be more rigid and less elastic than the unique triple‐helix structure of gelatin, which is known for its excellent viscoelastic properties. This more rigid, less flexible network could explain the lower hardness and springiness of the OFM samples. The tightly interconnected matrix would offer significant resistance to flow, contributing to the higher viscosity and more solid‐like rheological behavior observed in Figure 4. The gelatin triple helix is exceptionally effective at forming elastic films that stabilize air bubbles during aeration. The aquafaba‐gellan network, despite its strong binding energy, may not form films with the same elasticity. This could lead to a lower capacity for air incorporation or retention, resulting in the higher density observed in the OFM samples compared to the control. This connection between the molecular structure and the final foam properties helps to explain the observed differences in the final product.

### 3.9 Flow Behavior

Rheological measurements are crucial for the development, optimization, and processing of foods. These properties are instrumental in evaluating the sensory and textural characteristics of food products. The rheological properties of dispersions and emulsions serve as indicators of dispersed phase volume, size distribution, particle‐particle interaction, and particle deformability. In this context, microstructural properties play a significant role in determining the rheological behavior (Kavya et al. 2023). The rheological properties of gelatin and non‐gelatin confectionery products are essential for product development and characterization in the food industry.

The gelling point is a key parameter in products exhibiting gel‐like structures, as it represents the balance between deformation and mechanical strength (Ahmed 2017). Moreover, the rheological behavior of marshmallow‐type foam confectionery products influences their stability (Mardani et al. 2019). In marshmallow products, the fluidity of the mixture before aeration is a critical factor that impacts the aeration process. For instance, a mixture with a predominantly solid consistency will fail to retain sufficient air bubbles during aeration, which prevents the formation of a confectionery product with the desired density. The flow behavior characteristics of the marshmallow samples containing aquafaba and gellan gum (OFM) and the control samples containing gelatin (CM) are presented in Figure 4. Upon analyzing the data, it is evident that the sample with aquafaba and gellan gum is more viscous and more solid compared to the gelatin sample. The use of aquafaba and gellan gum in the marshmallow formulations reduced the foaming index and resulted in an increase in the viscosity of the samples. The flow behavior of marshmallow samples was analyzed using the Herschel‐Bulkley (HB) model, and the parameters are presented in Table 6. OFM possesses a significantly higher viscosity index *K* value than CM. This confirms that OFM exhibits a more viscous and solid structure compared to CM. OFM possesses a significantly higher yield stress compared to CM. The high *𝜎*_0_ value indicates that OFM requires a much greater force to begin yielding and better preserves its structure. This signifies the high level of mechanical strength, which is critical for foam stability. The use of aquafaba and gellan gum significantly increased the viscosity and solid character of the marshmallow mixture, proving that it provided high mechanical strength that would help maintain the structural integrity of the foam despite a lower foaming index.

**TABLE 6:** Flow behavior parameters of the OFM and CM marshmallow samples obtained from the Herschel‐Bulkley model.

Model	Parameters	CM	OFM
HB	*k* (Pa. s^n^)	0.836 ± 0,02^B^	3.948 ± 0,16^A^
	*σ*_0_ (Pa)	3.624 ± 0,19^B^	88.452 ± 33,26^A^
	*n*	1.07 ± 0,01^A^	0.88 ± 0,01^B^
	*R* ^2^	0.99	0.99

*Note:* Different capital superscript letters show the statistical difference between samples (*p* < 0.05).

Abbreviations: CM, control marshmallow; OFM, optimum formulation marshmallow.

### 3.10 Texture

Texture is one of the most important parameters influencing the acceptability of a product (Mardani et al. 2019). The results from the texture profile analysis of marshmallow samples are presented in Table 6. Hardness, the texture parameter that describes the force required to compress a foodstuff between the tongue and the palate, is also defined as the maximum force needed to physically compress food.

Marshmallow‐type confectionery products are complex due to their airy structure, and the texture of marshmallow samples is determined by macro‐scale properties such as hardness and the melting profile (Goktas et al. 2021). The sample containing aquafaba and gellan gum had a hardness value of 238.50 g, whereas the control sample had a value of 786.04 g. It was found that the hardness of the samples containing aquafaba and gellan gum was lower than that of the gelatin‐containing sample, which may be related to the water activity values of the samples. Marfil et al. (2012) used modified corn starch to improve the texture of gummy gels and reported that the addition of modified starch increased the hardness and opacity of the samples while decreasing their adhesiveness. They concluded that modified corn starch could be a suitable alternative for gummy gels.

Springiness refers to a product's ability to recover its original shape after compression (Yildiz et al. 2013). The use of aquafaba and gellan gum as gelatin substitutes resulted in a decrease in springiness values for the samples (*p* < 0.05). Resilience is defined as the recovery from deformation after an impact on the food material once the impact is removed (Ertaş and Doğruer 2010). The use of either gelatin or aquafaba and gellan gum as gelling agents did not significantly affect the resilience of the samples (*p* > 0.05). Cohesiveness, which reflects the strength of the internal bonds of a substance before compression, describes how easily the food is compressed between the teeth (Yildiz et al. 2013). No statistically significant differences were found in the cohesiveness values between the samples (*p* > 0.05). Therefore, it was determined that the combination of gellan gum and aquafaba was capable of forming a gel structure at least as effective as that of gelatin.

The formulation and processing conditions of confectionery products affect both the formation of the structure and the strength of the molecular structure, which in turn impacts the textural properties of the samples (Hartel et al. 2018). Chewability is defined by Ertaş and Doğruer (2010) as the time and energy required for chewing food until it is ready for swallowing. A statistical difference was observed in the chewability between the control marshmallow sample and the optimum marshmallow sample (*p* < 0.05). It is believed that the decrease in chewability is due to the fact that aquafaba and gellan gum form a structure that is less firm than gelatin. This finding is consistent with the hardness data. Bourne (2002) suggested that chewability may be closely related to resilience and, consequently, hardness. The significant reduction in hardness and chewiness in the OFM samples has several practical implications. From a consumer preference standpoint, whereas the OFM marshmallow does not replicate the firm, chewy bite of a traditional gelatin‐based product, its softer, more tender texture could appeal to a different consumer segment desiring a less resistant confectionery. This is supported by the sensory analysis, where the “overall liking” scores were comparable between the OFM and CM samples, indicating that this softer texture was still highly acceptable to the panelists. For handling and manufacturing, the lower hardness might present challenges, potentially requiring modified packaging to prevent deformation during transport and storage. However, this softer, more spreadable consistency could also be a significant advantage for new product applications, positioning the gelatin‐free formulation as an ideal filling for cakes and chocolate products or as a dessert topping, where a firm structure is not required.

### 3.11 Sensory

**FIGURE 5:**
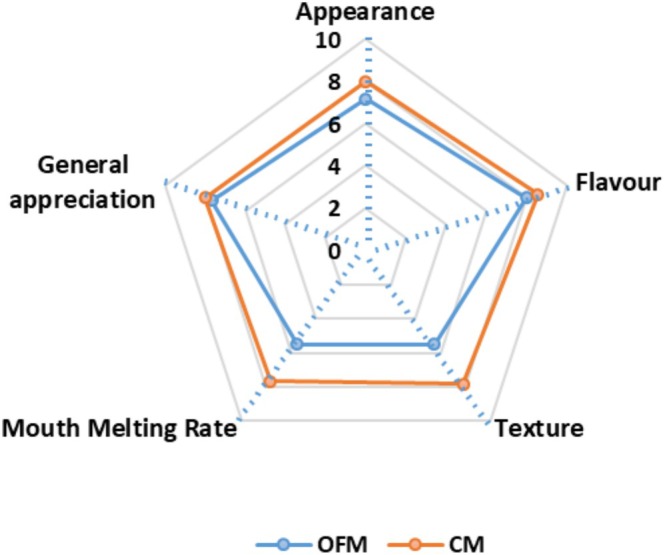
The sensory properties of OFM and CM samples (CM, control marshmallow, OFM, optimum formulation marshmallow).

The sensory evaluation results for marshmallow samples containing aquafaba and gellan gum (OFM) and those containing gelatin (CM) are presented in Figure 5. No significant differences were observed between the appearance, flavor, and general appreciation parameters of the OFM and CM samples (*p* > 0.05). However, a significant difference was found between the sensory texture and mouth‐melting rate parameters (*p* < 0.05). The melting rate of the CM samples was faster than that of the OFM samples (*p* < 0.05). These differences were found to be associated with the instrumental hardness parameter. Additionally, a correlation was observed between the density values of the samples and the sensory analysis results. Specifically, the sensory texture scores of the gelatin‐free samples, which had higher density values, were lower.

## 4 Conclusion

The optimum formulation for marshmallow‐type products was determined by evaluating the density and general appreciation results of samples made with plant‐based aquafaba and gellan gum as substitutes for gelatin (Table 7). In this optimal formulation, the usage rates of aquafaba and gellan gum were found to be 0.71% and 0.50%, respectively. It was observed that the density of the optimum marshmallow sample (without gelatin) was higher than that of the control marshmallow sample (containing gelatin). The combination of aquafaba and gellan gum as a gelatin substitute positively influenced the foaming properties of the marshmallow samples. Molecular docking analysis revealed that both proteins form highly stable complexes, with low binding energy indicating a more favorable and thermodynamically stable interaction during the docking process. However, the choice of hydrocolloid in the optimum samples caused significant changes in the structure, resulting in a decrease in the hardness value. The color of the products also changed, though these variations are unlikely to affect consumer expectations for standard marshmallow products. The sensory properties of the optimum marshmallow samples were similar to those of the control group containing gelatin. However, the desired firm and stable structure could not be achieved with aquafaba and gellan gum, leading to a more spreadable consistency. This result is further supported by the density values. The water activity value suggests that the shelf life of the new product may be shorter than that of a standard marshmallow. However, this disadvantage could be turned into an advantage by positioning gelatin‐free marshmallows as a cold confectionery product, or even using them as a filler in cakes or chocolate products. Furthermore, by incorporating the combination of aquafaba and gellan gum, it is possible to imbue confectionery products with functional properties, contributing to changes in eating habits. This study is the first to use aquafaba and gellan gum in marshmallow products, offering a valuable foundation for the development of future marshmallow‐type products containing plant‐based ingredients.

**TABLE 7:** The texture properties of OFM and CM samples.

Sample	Hardness (g)	Resilience (%)	Springness (%)	Cohesiveness (s)	Chewiness (g)
OFM	238.5 ± 143.3^B^	0.17 ± 0.03^B^	0.60 ± 0.31^A^	0.88 ± 0.01^A^	85.3 ± 1.58^B^
CM	786.0 ± 164.3^A^	0.47 ± 0.01^A^	0.51 ± 0.01^A^	1.12 ± 0.45^A^	390.0 ± 14.1^A^

*Note:* Different capital superscript letters show the statistical difference between samples (*p* < 0.05).

Abbreviations: CM, control marshmallow; OFM, optimum formulation marshmallow.

## Author Contributions

**İbrahim Palabiyik:** conceptualization, methodology, writing – review and editing, supervision. **Serpil Pekdogan Goztok:** writing – review and editing, visualization. **Deniz D. A. Kamer:** formal analysis, writing – review and editing. **Elif Ersahin Telli:** methodology, formal analysis. **Nevzat Konar:** writing – review and editing.

## Funding

This study was granted by Scientific Research Projects CoordinationUnit of Tekirdag Namık Kemal University. Project number: NKUBAP.03.YL.22.429.

## Conflicts of Interest

The authors declare no conflicts of interest.

## Supporting information


**Table S1:** Analysis of variance (ANOVA) summary table for the effect of aquafaba (A) and gellan gum (B) concentrations.

## Data Availability

The data that support the findings of this study are available from the corresponding author upon reasonable request.

## References

[fsn371898-bib-0001] Ahmed, J. 2017. “Rheological Properties of Gelatin and Advances in Measurement.” In Advances in Food Rheology and Its Applications, 1th ed., 377–404. Woodhead Press. 10.1016/B978-0-08-100431-9.00015-2.

[fsn371898-bib-0002] Altınok, E. , I. Palabiyik , R. Gunes , O. S. Toker , N. Konar , and S. Kurultay . 2020. “Valorisation of Grape By‐Products as a Bulking Agent in Soft Candies: Effect of Particle Size.” Lwt 118: 108776. 10.1016/j.lwt.2019.108776.

[fsn371898-bib-0003] Aslan, M. , and N. Ertaş . 2020. “Possibility of Using Chickpea Aquafabas Egg Replacer in Traditional Cake Formulation.” Harran Tarım ve Gıda Bilimleri Dergisi 24, no. 1: 1–8. 10.29050/harranziraat.569397.

[fsn371898-bib-0004] Atik, D. S. 2021. “Jelatin bazlı yumuşak şekerlerin erime özelliklerinin reometre temelli yeni metot ile belirlenmesi.” Doktora Tezi, Tekirdağ Namık Kemal Üniversitesi, Fen Bilimleri Enstitüsü, Tekirdağ.

[fsn371898-bib-0005] Azam, S. S. , and S. W. Abbasi . 2013. “Molecular Docking Studies for the Identification of Novel Melatoninergic Inhibitors for Acetylserotonin‐O‐Methyltransferase Using Different Docking Routines.” Theoretical Biology and Medical Modelling 10: 1–16. 10.1186/1742-4682-10-63. 24156411 PMC3819668

[fsn371898-bib-0006] Bourne, M. 2002. Food Texture and Viscosity: Concept and Measurement. 2th ed. Academic Press.

[fsn371898-bib-0007] Buhl, T. F. , C. H. Christensen , and M. Hammershøj . 2019. “Aquafaba as an Egg White Substitute in Food Foams and Emulsions: Protein Composition and Functional Behavior.” Food Hydrocolloids 96, no. 8: 354–364. 10.1016/j.foodhyd.2019.05.041.

[fsn371898-bib-0008] Campbell, G. M. , and E. Mougeot . 1999. “Creation and Characterisation of Aerated Food Products.” Trends in Food Science & Technology 10, no. 9: 283–296. 10.1016/S0924-2244(00)00008-X.

[fsn371898-bib-0009] Covaliov, E. , N. Suhodol , A. Chirsanova , T. Capcanari , C. Grosu , and R. Siminiuc . 2021. “Effect of Grape Skin Powder Extract Addition on Functional and Physicochemical Properties of Marshmallow.” Ukrainian Food Journal 10, no. 2: 333–345. 10.24263/2304-974X-2021-10-2-10.

[fsn371898-bib-0010] Crawford, K. , C. Tyl , and W. Kerr . 2023. “Evaluation of Processing Conditions and Hydrocolloid Addition on Functional Properties of Aquafaba.” Food 12, no. 4: 775. 10.3390/foods12040775. PMC9956225 36832848

[fsn371898-bib-0011] Dev, M. J. , R. G. Warke , G. M. Warke , G. B. Mahajan , T. A. Patil , and R. S. Singhal . 2022. “Advances in Fermentative Production, Purification, Characterization and Applications of Gellan Gum.” Bioresource Technology 359, no. 231: 127498. 10.1016/j.biortech.2022.127498. 35724911

[fsn371898-bib-0012] Eddy, S. , and F. Editya . 2020. “The Effect of Concentrations of *Ephinephelus* sp. Skin Gelatin on the Quality of Halal Marshmallows.” Russian Journal of Agricultural and Socio‐Economic Sciences 97, no. 1: 120–125. 10.18551/rjoas.2020-01.15.

[fsn371898-bib-0013] Erdem, N. , N. G. Taş , T. Kocadağlı , and V. Gökmen . 2023. “Modelling of Perceived Sweetness in Biscuits Based on Sensory Analysis as a New Tool to Evaluate Reformulation Performance in Sugar Reduction Studies.” Food Chemistry 425, no. 3: 136490. 10.1016/j.foodchem.2023.136490. 37276663

[fsn371898-bib-0015] Ertaş, N. , and Y. Doğruer . 2010. “Besinlerde Tekstür.” Erciyes Üniversitesi Veteriner Fakültesi Dergisi 7, no. 1: 35–42.

[fsn371898-bib-0016] Ghendov‐Moșanu, A. 2018. “The Use of Dog‐Rose Hips ( *Rosa canina* ) Fruits in the Production of Marshmallow Type Candy.” Food and Environment Safety 27, no. 1: 59–65.

[fsn371898-bib-0017] Gok, S. , O. S. Toker , I. Palabiyik , and N. Konar . 2020. “Usage Possibility of Mannitol and Soluble Wheat Fiber in Low Calorie Gummy Candies.” LWT‐ Food Science and Technology 128, no. 1: 109531. 10.1016/j.lwt.2020.109531.

[fsn371898-bib-0018] Goktas, H. , N. Konar , O. Sagdic , and O. S. Toker . 2021. “Investigation Effects of Inulin Degree of Polymerization on Compound Chocolate Quality.” Journal of Food Processing and Preservation 45, no. 11: e15766. 10.1111/jfpp.15766.

[fsn371898-bib-0019] Gunes, R. , I. Palabiyik , N. Konar , and O. S. Toker . 2022. “Soft Confectionery Products: Quality Parameters, Interactions With Processing and Ingredients.” Food Chemistry 385, no. 6: 132735. 10.1016/j.foodchem.2022.132735. 35318175

[fsn371898-bib-0020] Han, X. , Y. Zhao , S. Mao , et al. 2022. “Effects of Different Amounts of Corn Silk Polysaccharide on the Structure and Function of Peanut Protein Isolate Glycosylation Products.” Food 11, no. 15: 2214. 10.3390/foods11152214. PMC9330836 35892799

[fsn371898-bib-0021] Hartel, R. W. , H. Joachim , and R. Hofberger . 2018. Confectionery Science and Technology. 1th ed, 85–124. Springer.

[fsn371898-bib-0022] He, Y. , V. Meda , M. J. Reaney , and R. Mustafa . 2021. “Aquafaba, a New Plant‐Based Rheological Additive for Food Applications.” Trends in Food Science & Technology 111, no. 2: 27–42. 10.1016/j.tifs.2021.02.035.

[fsn371898-bib-0023] Hutchings, J. B. 2011. Food Colour and Appearance. 1th ed. Springer Science & Business Media.

[fsn371898-bib-0024] Jindal, N. , and J. S. Khattar . 2018. “Microbial Polysaccharides in Food Industry.” In Biopolymers for Food Design, 1th ed., 95–123. Academic Press.

[fsn371898-bib-0025] Karim, A. A. , and R. Bhat . 2008. “Gelatin Alternatives for the Food Industry: Recent Developments, Challenges and Prospects.” Trends in Food Science & Technology 19, no. 12: 644–656. 10.1016/j.tifs.2008.08.001.

[fsn371898-bib-0026] Kavya, M. , A. R. Jacob , and P. Nisha . 2023. “Pectin Emulsions and Emulgels: Bridging the Correlation Between Rheology and Microstructure.” Food Hydrocolloids 143, no. 1: 108868. 10.1016/j.foodhyd.2023.108868.

[fsn371898-bib-0027] Kazantsev, E. V. , N. B. Kondratev , O. S. Rudenko , N. A. Petrova , and I. A. Belova . 2022. “Formation of a Foamy Structure of Confectionery Pastille Products.” Food Systems 5, no. 1: 64–69. 10.21323/2618-9771-2022-5-1-64-69.

[fsn371898-bib-0028] Klinjapo, R. , and W. Krasaekoopt . 2018. “Microencapsulation of Color and Flavor in Confectionery Products.” In Natural and Artificial Flavoring Agents and Food Dyes, 2th ed., 457–494. Academic Press. 10.1016/B978-0-12-811518-3.00014-4.

[fsn371898-bib-0029] Manuel, J. B. J. , R. M. Jesús , H. L. Erasmo , A. C. Andrés , M. V. V. Manuel , and H. S. Betsabé . 2022. “Application of Differential Scanning Calorimetry to Dairy Foods.” In Dairy Foods, 1th ed., 233–260. Woodhead Press. 10.1016/B978-0-12-820478-8.00004.

[fsn371898-bib-0030] Mardani, M. , S. Yeganehzad , N. Ptichkina , et al. 2019. “Study on Foaming, Rheological and Thermal Properties of Gelatin‐Free Marshmallow.” Food Hydrocolloids 93, no. 2: 335–341. 10.1016/j.foodhyd.2019.02.033.

[fsn371898-bib-0031] Marfil, P. H. , A. C. Anhê , and V. R. Telis . 2012. “Texture and Microstructure of Gelatin/Corn Starch‐Based Gummy Confections.” Food Biophysics 7, no. 3: 236–243. 10.1007/s11483-012-9262-3.

[fsn371898-bib-0056] Meng, Y. , Z. Wei , and C. Xue . 2023. “Deciphering the Interaction Mechanism and Binding Mode Between Chickpea Protein Isolate and Flavonoids Based on Experimental Studies and Molecular Simulation.” Food Chemistry 429: 136848. 10.1016/j.foodchem.2023.136848. 37454615

[fsn371898-bib-0057] Morris, C. J. , and D. D. Corte . 2021. “Using Molecular Docking and Molecular Dynamics to Investigate Protein‐Ligand Interactions.” Modern Physics Letters B 35, no. 08: 2130002. 10.1142/s0217984921300027.

[fsn371898-bib-0032] Morris, G. M. , R. Huey , W. Lindstrom , et al. 2009. “AutoDock4 and AutoDockTools4: Automated Docking With Selective Receptor Flexibility.” Journal of Computational Chemistry 30, no. 16: 2785–2791. 10.1002/jcc.21256. 19399780 PMC2760638

[fsn371898-bib-0033] Mustafa, R. , Y. He , Y. Y. Shim , and M. J. Reaney . 2018. “Aquafaba, Wastewater From Chickpea Canning, Functions as an Egg Replacer in Sponge Cake.” International Journal of Food Science & Technology 53, no. 10: 2247–2255. 10.1111/ijfs.13813.

[fsn371898-bib-0034] Nguyen, T. M. N. , T. P. Nguyen , G. B. Tran , and P. T. Q. Le . 2020. “Effect of Processing Methods on Foam Properties and Application of Lima Bean ( *Phaseolus lunatus* L.) Aquafaba in Eggless Cupcakes.” Journal of Food Processing and Preservation 44, no. 11: e14886. 10.1111/jfpp.14886.

[fsn371898-bib-0035] Pauling, L. , and R. B. Corey . 1951. “The Pleated Sheet, a New Layer Configuration of Polypeptide Chains.” Proceedings of the National Academy of Sciences 37, no. 5: 251–256. 10.1073/pnas.37.5.251. PMC1063350 14834147

[fsn371898-bib-0036] Peinado, I. , U. Lesmes , A. Andrés , and J. D. McClements . 2010. “Fabrication and Morphological Characterization of Biopolymer Particles Formed by Electrostatic Complexation of Heat Treated Lactoferrin and Anionic Polysaccharides.” Langmuir 26, no. 12: 9827–9834. 10.1021/la1001013. 20229991

[fsn371898-bib-0037] Pekdogan Goztok, S. , R. Gunes , O. S. Toker , I. Palabiyik , and N. Konar . 2022. “Investigation of the Use of Various Fruit Juice Concentrates Instead of Corn Syrup in Marshmallow Type Products: A Preliminary Study.” International Journal of Gastronomy and Food Science 30, no. 2: 100616. 10.1016/j.ijgfs.2022.100616.

[fsn371898-bib-0055] Periche, A. , A. Heredia , I. Escriche , A. Andrés , and M. L. Castelló . 2015. “Potential Use of Isomaltulose to Produce Healthier Marshmallows.” LWT – Food Science and Technology 62, no. 1: 605–612. 10.1016/j.lwt.2014.12.024.

[fsn371898-bib-0038] Periche, Á. , M. L. Castelló , A. Heredia , and I. Escriche . 2016. “ *Stevia rebaudiana* , Oligofructose and Isomaltulose as Sugar Replacers in Marshmallows: Stability and Antioxidant Properties.” Journal of Food Processing and Preservation 40, no. 4: 724–732. 10.1111/jfpp.12653.

[fsn371898-bib-0039] Piliugina, І. , M. Artamonova , N. Murlykina , and О. Shidakova‐Kamenyuka . 2019. “Study of the Foamıng Propertıes of Gelatın With Solubılızed Substances for the Productıon of Marshmallows.” Food Science and Technology 13, no. 1: 90–97. 10.15673/fst.v13i1.1335.

[fsn371898-bib-0040] Rahman, M. S. 2019. “Water Activity and Glass Transition of Foods.” In Reference Module in Food Science, 2nd ed., 1–10. Elsevier Press.

[fsn371898-bib-0041] Raikos, V. , H. Hayes , and H. Ni . 2020. “Aquafaba From Commercially Canned Chickpeas as Potential Egg Replacer for the Development of Vegan Mayonnaise: Recipe Optimisation and Storage Stability.” International Journal of Food Science & Technology 55, no. 5: 1935–1942. 10.1111/ijfs.14427.

[fsn371898-bib-0042] Sablani, S. S. , S. Kasapis , and M. S. Rahman . 2007. “Evaluating Water Activity and Glass Transition Concepts for Food Stability.” Journal of Food Engineering 78, no. 1: 266–271. 10.1016/j.jfoodeng.2005.09.025.

[fsn371898-bib-0043] Şahin, A. 2021. “Farklı formlarda geliştirilen glütensiz galetaların besin değerleri, duyusal analizleri ve satın alma niyeti açısından değerlendirilmesi.” Yüksek lisans tezi, Kocaeli Üniversitesi Sosyal Bilimler Enstitüsü, Kocaeli.

[fsn371898-bib-0044] Samira, Y. 2018. “Study of the Stability of Foam and Viscoelastic Properties of Marshmallow Without Gelatin.” Foods and Raw Materials 6, no. 1: 90–98.

[fsn371898-bib-0045] Sani, M. S. A. , A. M. Ismail , A. Azid , and M. S. Samsudin . 2021. “Establishing Forensic Food Modellings for Authentication and Quantification of Porcine Adulterant in Gelatine and Marshmallow.” Food Control 130, no. 3: 108350. 10.1016/j.foodcont.2021.108350.

[fsn371898-bib-0046] Sheng, Y. , S. Lu , M. Xu , X. Wu , and C. Li . 2016. “Effect of Xanthan Gum on the Performance of Aqueous Film‐Forming Foam.” Journal of Dispersion Science and Technology 37, no. 11: 1664–1670. 10.1080/01932691.2015.1124341.

[fsn371898-bib-0047] Subbiah, B. , U. K. Blank , and K. R. Morison . 2020. “A Review, Analysis and Extension of Water Activity Data of Sugars and Model Honey Solutions.” Food Chemistry 326: 126981. 10.1016/j.foodchem.2020.126981. 32416420

[fsn371898-bib-0048] Sworn, G. , G. R. Sanderson , and W. Gibson . 1995. “Gellan Gum Fluid Gels.” Food Hydrocolloids 9, no. 4: 265–271. 10.1016/S0268-005X(09)80257-9.

[fsn371898-bib-0049] Tan, J. M. , and M. H. Lim . 2008. “Effects of Gelatine Type and Concentration on the Shelf‐Life Stability and Quality of Marshmallows.” International Journal of Food Science & Technology 43, no. 9: 1699–1704. 10.1111/j.1365-2621.2008.01756.x.

[fsn371898-bib-0050] Terashima, Y. 2022. “Thermal Study on Cotton Candy by Differential Scanning Calorimetry.” Chemical Physics Letters 805, no. 4: 139953. 10.1016/j.cplett.2022.139953.

[fsn371898-bib-0051] Tufaro, D. , and C. Cappa . 2023. “Chickpea Cooking Water (Aquafaba): Technological Properties and Application in a Model Confectionery Product.” Food Hydrocolloids 136: 108231. 10.1016/j.foodhyd.2022.108231.

[fsn371898-bib-0052] Xiao, H. , S. Wang , W. Xu , et al. 2020. “The Study on Starch Granules by Using Darkfield and Polarized Light Microscopy.” Journal of Food Composition and Analysis 92: 103576. 10.1016/j.jfca.2020.103576.

[fsn371898-bib-0053] Yildiz, Ö. , B. Yurt , A. Baştürk , et al. 2013. “Pasting Properties, Texture Profile and Stress Relaxation Behavior of Wheat Starch/Dietary Fiber Systems.” Food Research International 53, no. 1: 278–290. 10.1016/j.foodres.2013.04.018.

[fsn371898-bib-0054] Yilmaz, O. Ş. , T. Gümüş , G. B. Kaynarca , and D. D. A. Kamer . 2023. “Effect of Addition of Different Gums on the Technological and Rheological Properties of Fish Gelatin.” Tekirdağ Ziraat Fakültesi Dergisi 20, no. 3: 663–676.

