# Supplementary material for: Optimization and Molecular Simulation of Gelatin‐Free Marshmallow Formulation Using Gellan Gum and Aquafaba
Source: Food Sci Nutr. 2026 May 24;14(5):e71898. doi: 10.1002/fsn3.71898 (PMC13239071; doi:10.1002/fsn3.71898)
Supplement: Supplementary file 1 — Table S1: Analysis of variance (ANOVA) summary table for the effect of aquafaba (A) and gellan gum (B) concentrations. [file FSN3-14-e71898-s001.docx]

Table S1. Analysis of Variance (ANOVA) Summary Table for the Effect of Aquafaba (A) and Gellan Gum (B) Concentrations

| Response | Model type | Source | SS | df | MS | F-value | p-value |
| --- | --- | --- | --- | --- | --- | --- | --- |
| Appearance | Linear | Model | 22.56 | 2 | 11.28 | 6.24 | 0.0278 |
|  |  | A–aquafaba | 0.1350 | 1 | 0.1350 | 0.0747 | 0.7925 |
|  |  | B–gellan | 22.43 | 1 | 22.43 | 12.41 | 0.0097 |
|  |  | Residual | 12.65 | 7 | 1.81 | – | – |
|  |  | Lack of Fit | 11.81 | 6 | 1.97 | 2.33 | 0.4634 |
|  |  | Pure Error | 0.8450 | 1 | 0.8450 | – | – |
| Texture | Quadratic | Model | 13.40 | 3 | 4.47 | 5.01 | 0.0450 |
|  |  | A | 0.4267 | 1 | 0.4267 | 0.4787 | 0.5149 |
|  |  | B | 12.91 | 1 | 12.91 | 14.48 | 0.0089 |
|  |  | AB | 0.0625 | 1 | 0.0625 | 0.0701 | 0.8000 |
|  |  | Residual | 5.35 | 6 | 0.8914 | – | – |
|  |  | Lack of Fit | 5.34 | 5 | 1.07 | 213.73 | 0.0519 |
|  |  | Pure Error | 0.0050 | 1 | 0.0050 | – | – |
| Flavour | 2FI | Model | 8.20 | 3 | 2.73 | 2.68 | 0.1406 |
|  |  | A | 0.0017 | 1 | 0.0017 | 0.0016 | 0.9691 |
|  |  | B | 7.71 | 1 | 7.71 | 7.55 | 0.0334 |
|  |  | AB | 0.4900 | 1 | 0.4900 | 0.4802 | 0.5143 |
|  |  | Residual | 6.12 | 6 | 1.02 | – | – |
|  |  | Lack of Fit | 6.04 | 5 | 1.21 | 15.11 | 0.1928 |
|  |  | Pure Error | 0.0800 | 1 | 0.0800 | – | – |
| Adhesiveness | 2FI | Model | 6.46 | 3 | 2.15 | 4.89 | 0.0474 |
|  |  | A | 1.71 | 1 | 1.71 | 3.87 | 0.0967 |
|  |  | B | 4.51 | 1 | 4.51 | 10.22 | 0.0187 |
|  |  | AB | 0.2500 | 1 | 0.2500 | 0.5670 | 0.4800 |
|  |  | Residual | 2.65 | 6 | 0.4409 | – | – |
|  |  | Lack of Fit | 2.64 | 5 | 0.5281 | 105.63 | 0.0737 |
|  |  | Pure Error | 0.0050 | 1 | 0.0050 | – | – |
| Density | Quadratic | Model | 0.2735 | 5 | 0.0547 | 61.72 | 0.0007 |
|  |  | A | 0.1980 | 1 | 0.1980 | 223.42 | 0.0001 |
|  |  | B | 0.0561 | 1 | 0.0561 | 63.26 | 0.0014 |
|  |  | AB | 0.0049 | 1 | 0.0049 | 5.53 | 0.0784 |
|  |  | A² | 0.0144 | 1 | 0.0144 | 16.25 | 0.0157 |
|  |  | B² | 0.0001 | 1 | 0.0001 | 0.1088 | 0.7581 |
|  |  | Residual | 0.0035 | 4 | 0.0009 | – | – |
|  |  | Lack of Fit | 0.0031 | 3 | 0.0010 | 2.29 | 0.4438 |
|  |  | Pure Error | 0.0005 | 1 | 0.0005 | – | – |
